# Filaria Sanguinis Hominis

**Published:** 1882-03-20

**Authors:** 


### FILARIA SANGUINIS HOMINIS.

Dr. Myers, of Takow, Formosa, has recently added to our knowledge of the *Filaria sanguinis hominis* the interesting results of numerous observations made by himself upon filaria-infected patients. He instituted these examinations, he tells us, at the request of Dr. Patrick Manson, with the object in view of investigating, and, if possible, confirming, the discoveries and observations made by that gentleman. He endeavored, therefore, to determine the proportion of patients affected, noting the external manifestations present,—*e. g.*, elephantiasis, lymph-scrotum, etc.; the periodicity of appearance and disappearance of the embryos; the cause of the diurnal disappearance, the centre of congregation, if any; the effects of "infilariation" of the monkey. After examining a large number of patients, he succeeded in finding but three in whose blood the filaria appeared, neither of whom, however, presented any appearnce of elephantiasis or lymph-scrotum. One of these, a boatman (born in Amoy, To Ah, by name), he succeeded in prevailing upon to submit to daily puncturing of the skin for obtaining specimens of blood for microscopic examination. This good-natured boatman, To Ah, was placed under a large mosquito-net, and mosquitoes from all parts of the island were allowed to feed upon him nightly. A trough was suspended within the net, and filled with water, upon which the mosquitoes deposited their ova. This water was given to monkeys to drink, no other fluid being allowed them except that contained in the bananas on which they were fed. Frequent examinations of the blood of To Ah showed the presence of the filaria at night and their absence during a greater part of the day. The temperature, taken at each observation, generally rose slightly with the appearance of the embryos, and fell again with their disappearnce. The mosquitoes fed upon To Ah were also subjected to microscopic examination, the half-digested remains of the embryos being all that could be found. The attempts to filariate the monkeys by allowing them to be bitten by these mosquitoes, and by compelling them to drink water upon which the ova of these mosquitoes had been deposited, both failed, the monkeys eventually dying of tuberculosis, a disease common among those kept in captivity.

Dr. Myers expresses the opinion that the mosquitoes which play the part of host in the embryo during its development must be of a different species from those found in Formosa, which digest the embryo instead of nurturing it; and to this fact he attributes the apparent immunity of the natives of that island from the filaria.

In concluding his observations, Dr. Myers made a number of experiments npon the filaria with various drugs, such as arsenious acid, salicylic acid, bisulphate of quinia, and santonin, and was surprised to find that a comparatively large quantity was required to destroy it—sufficient, indeed, to destroy the patient as well.

Dr. Myers's paper is equally valuable and interesting, and reflects great credit upon its author for the untiring zeal and perseverance with which he has followed up the subject. We would suggest that the experiment be made of changing the hours of rest and maximum light by requiring the patient to sleep in a darkened room during the day, and to note the effect upon the appearance and disappearance of the embryos.—*Med. Times.*

